# Randomized controlled trial of nasogastric tube use after esophagectomy: study protocol for the kinetic trial

**DOI:** 10.1093/dote/doae010

**Published:** 2024-02-16

**Authors:** Jakob Hedberg, Magnus Sundbom, David Edholm, Eirik Kjus Aahlin, Eva Szabo, Fredrik Lindberg, Gjermund Johnsen, Dag Tidemann Førland, Jan Johansson, Joonas H Kauppila, Lars Bo Svendsen, Magnus Nilsson, Mats Lindblad, Pernilla Lagergren, Michael Hareskov Larsen, Oscar Åkesson, Per Löfdahl, Tom Mala, Michael Patrick Achiam

**Affiliations:** Department of Surgical Sciences, Uppsala University, Uppsala, Sweden; Department of Surgical Sciences, Uppsala University, Uppsala, Sweden; Department of Surgery in Linköping and Department of Biomedical and Clinical Sciences, Linköping University, Linköping, Sweden; Department of GI and HPB Surgery, Institute of Clinical Medicine, University of Tromsø, Tromsø, Norway; Department of GI and HPB Surgery, University Hospital of Northern Norway, Tromsø, Norway; Department of Surgery, Örebro University, Örebro, Sweden; Department of Surgical and Perioperative Sciences Surgery, Umeå University, Umeå, Sweden; Department of Gastrointestinal Surgery, Norwegian University of Science and Technology, Trondheim, Norway; Department of Pediatric and Gastrointestinal Surgery, Oslo University Hospital, Oslo, Norway; Department of Surgery, Skane University Hospital, Lund, Sweden; Department of Surgery, University of Oulu and Oulu University Hospital, Oulu, Finland; Department of Surgery and Transplantation, Copenhagen University Hospital, Copenhagen, Denmark; Department of Clinical Science, Intervention and Technology, Division of Surgery, Karolinska Institutet, Stockholm, Sweden; Nuffield Department of Surgical Sciences, University of Oxford, Oxford, UK; Department of Clinical Science, Intervention and Technology, Division of Surgery, Karolinska Institutet, Stockholm, Sweden; Department of Molecular Medicine and Surgery, Karolinska Institutet, Stockholn, Sweden; Department of Surgery and Cancer, Imperial College London, London, UK; Department of Surgery, Odense University Hospital, Odense, Denmark; Department of Surgery, Skane University Hospital, Lund, Sweden; Department of Surgery, Sahlgrenska University Hospital, Gothenburg, Sweden; Department of Pediatric and Gastrointestinal Surgery, Oslo University Hospital, Oslo, Norway; Department of Surgery and Transplantation, Copenhagen University Hospital, Copenhagen, Denmark; Department of Clinical Medicine, University of Copenhagen, Copenhagen, Denmark

**Keywords:** complications, esophagectomy, surgery, trials

## Abstract

Esophagectomy is a complex and complication laden procedure. Despite centralization, variations in perioparative strategies reflect a paucity of evidence regarding optimal routines. The use of nasogastric (NG) tubes post esophagectomy is typically associated with significant discomfort for the patients. We hypothesize that immediate postoperative removal of the NG tube is non-inferior to current routines. All Nordic Upper Gastrointestinal Cancer centers were invited to participate in this open-label pragmatic randomized controlled trial (RCT). Inclusion criteria include resection for locally advanced esophageal cancer with gastric tube reconstruction. A pretrial survey was undertaken and was the foundation for a consensus process resulting in the Kinetic trial, an RCT allocating patients to either no use of a NG tube (intervention) or 5 days of postoperative NG tube use (control) with anastomotic leakage as primary endpoint. Secondary endpoints include pulmonary complications, overall complications, length of stay, health related quality of life. A sample size of 450 patients is planned (Kinetic trial: https://www.isrctn.com/ISRCTN39935085). Thirteen Nordic centers with a combined catchment area of 17 million inhabitants have entered the trial and ethical approval was granted in Sweden, Norway, Finland, and Denmark. All centers routinely use NG tube and all but one center use total or hybrid minimally invasive-surgical approach. Inclusion began in January 2022 and the first annual safety board assessment has deemed the trial safe and recommended continuation. We have launched the first adequately powered multi-center pragmatic controlled randomized clinical trial regarding NG tube use after esophagectomy with gastric conduit reconstruction.

## INTRODUCTION

Esophageal cancer is among the most common cancers in the world with more than 600,000 new cases worldwide in 2020.^1^ While important progress has been made by the addition of perioperative oncological treatment, improving survival,^2–5^ surgical resection remains the mainstay for curative treatment of esophageal and gastroesophageal junctional cancer. Yet, this surgery is associated with high rates of complications, which may affect both long-term survival and cancer recurrence.^6–9^ This has led to a firm adherence to standardized surgical procedures and perioperative management protocols implemented to prevent and reduce the potential consequences of complications.^10^ Nevertheless, the perioperative treatment strategies and protocols differ across institutions. The evidence for several of the guidelines adhered to are limited. This is evident in the heterogeneity in the literature on the use of pyloric drainage, feeding jejunostomy, and nasogastric (NG) tube placement.^11–14^ A recent study focused on the emptying of the gastric conduit, showed that only 7% of patients had severely delayed emptying of the gastric conduit on Day 3 and no increased risk was seen in this group.^15^ NG tube placement is reported uncomfortable by many patients as the most disturbing part of the postoperative care,^16^ and potentially interfering with enhanced recovery strategies post-surgery.

In the Nordic countries (Sweden, Norway, Denmark, Finland, and Iceland), the treatment of esophageal cancer is largely centralized in Upper Gastrointestinal Cancer (UGC) centers in University Hospitals and a research network has been established. We have launched a multi-center pragmatic randomized controlled clinical trial (RCT) to investigate if immediate post-operative removal of the NG tube is non-inferior compared to current routines of several days of NG tube use, primarily regarding the risk of postoperative anastomotic leak.

## METHODS

All 21 University Hospitals with a UGC center in the Nordic countries were contacted regarding this prospective, randomized trial (Kinetic trial: https://www.isrctn.com/ISRCTN39935085) investigating NG-tube decompression after resectional surgery for esophageal and gastroesophageal junctional cancer. A questionnaire was distributed to participating centers regarding surgical volumes and practices, including the mean annual number of esophagectomies in 2021, preferred surgical access, and routine use of pyloric drainage and feeding jejunostomy. In addition, the current local perioperative management protocols of using a NG-tube, X-ray/CT evaluation of the anastomotic integrity, and the postoperative start of liquid diet were enquired.

Based on the response to this questionnaire, a consensus process was started and the participating centers all agreed to the following.

The patients are randomized after completed resection to (a) intervention, immediate postoperative NG tube removal/no tube or (b) control, 5 days with NG tube no less than Ch16 and suction according to local routines (passive siphon or intermittent active suction). The tube can be left for more than 5 days if the output exceeds 300 mL or if deemed indicated by responsible surgeon. Any deviations from planned NG tube use (5 days) are recorded in the electronic Case Report Form (eCRF).

Inclusion criteria: Histopathologically confirmed esophageal or gastroesophageal junctional cancer in locally advanced stages (cT1a N+ or cT1b-4a any N; M0) considered technically resectable by the local tumor board; Age ≥ 18 years; Planned for esophagectomy with gastric conduit reconstruction and written informed consent.

Exclusion criteria: No resection performed (reason specified); alternative reconstruction method used (Roux-limb/colonic interponate); surgeon choosing to leave NG-tube (reason specified); no ability to understand the study in terms of risk and benefits (including language difficulties).

**Fig. 1:**
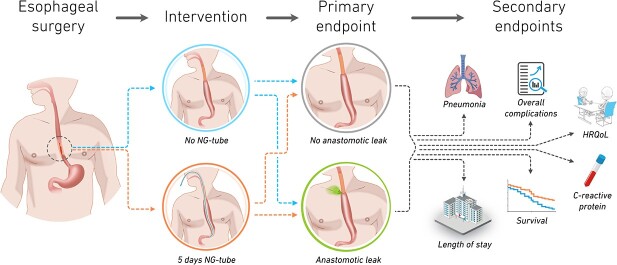
In this Nordic multi-center trial, we will use a controlled study design to provide the first high-level evidence regarding complications associated with nasogastric tube use after esophageal resection for cancer. Copyright Jakob Hedberg.

The primary endpoint is anastomotic leakage defined in accordance with Low *et al.*^17^ and secondary endpoints are pneumonia according to Seesing *et al.*^18^; postoperative complications >3a according to Clavien–Dindo^19^; respiratory failure requiring invasive or non-invasive respiratory support; C-reactive protein Day 1–7; length of stay; length of ICU-stay; reintervention with NG tube decompression and vomiting. In addition, investigating health related quality of life with structured interviews is planned (Figure 1).

An online randomization module has been created and permuted block randomization with stratification for sex, anastomosis site (neck or thorax) neoadjuvant treatment (y/n), and center is performed in the operating theatre before waking the patient. On Day 7, a Chest CT with peroral contrast is performed. No extra visits are planned for this trial and follow up data is entered in an eCRF including all events up to 6 weeks after surgery. Aside from this, local traditions on postoperative X-ray examinations and other postoperative routines are not affected by the trial.

Monitoring visits to the trial centers are performed continuously by good clinical practice trained personnel.

### Statistics

Anastomotic leak is seen in between 5% and 30% after esophagectomy. In a recent Scandinavian trial (performed by the network), the leak-rate was 18%.^20^ With a prevalence of 17%, a significance level (α) of 5%, 80% power (β), and a non-inferiority threshold (delta) of 9% would require 216 patients in each group to reject the null-hypothesis (intervention inferior). In order to have a margin for unexpected withdrawals, we plan for randomization of 225 patients to each group. Included centers perform together 420 resections per year. The representativeness of the total study sample will be investigated by comparing all eligible patients for screening with national quality registries.

All outcomes measures will be analyzed using the intention to treat principle where all patients randomized to a certain group will be followed and analyzed irrespective of the actual treatment, offering unbiased assessments of treatment efficacy. In addition, per-protocol analyses will also be performed.

The primary objective, non-inferiority of the experimental arm (no NG tube), will be assessed overall and with stratified (women aged <70 years, women aged ≥70 years, men aged <70 years, and men aged ≥70 years) Miettinen–Nurminen two-sided 90% confidence interval (CI) for the difference in proportions and one-sided *P*-value for non-inferiority. Non-inferiority will be considered shown if *P* < 0.05 (one-sided).

Non-inferiority will be established on the absolute scale for the sample proportions. To estimate the treatment effect on a scale transferable to individual patients, we will use logistic regression adjusted for sex, age (as a linear covariate on the log-odds scale), level of anastomosis (chest or neck), comorbidity according to the American Society for Anesthesiology (ASA score), (<2/≥2), clinical tumor stage, and neoadjuvant treatment (y/n). The adjusted odds ratio will be presented with two-sided 95% CI and the 2-sided *P*-value for no difference in odds ratio. Sensitivity analyses including unadjusted analysis will also be performed.

Subgroup analyses of the primary endpoint will be performed by introducing subgroup indicators (if not already included) and a treatment-subgroup interaction term in the logistic regression model, excluding any patients not possible to classify. These subgroups will include age (above/below 70 years), sex (male/female), and level of anastomosis (chest/neck). Estimates of treatment differences will be presented as subgroup-specific odds ratios with 95% CIs and interaction *P*-values.

Secondary endpoints will be analyzed without adjustment for multiplicity. Data monitoring and safety analysis of overall complications has been undertaken by an independent data Safety and Monitoring Committee after one year of patient inclusion. The committee deemed the trial to be safe to continue. It also demonstrated an overall frequency of the primary outcome within the expected range and no missing data for the primary outcome.

The trial has been approved by the relevant ethical oversight authorities in Sweden (Dnr 2021-03761), Norway (Nr 256722), Finland (dnr 85/2021/266§), and Denmark (Jnr H21069333) and annual reviews of an international independent data safety and monitoring committee will be performed.

## RESULTS

A total of 13 of the 21 eligible centers in four countries have entered the trial to date. Surgical volumes varied between 10 and 110 annually with a median of 22.5 esophagectomies per year, which amounts to 2.5 esophagectomies per 100,000 inhabitants in the 17 million inhabitant catchment area of the included centers.

Before entering the trial, all centers routinely used NG tube after esophagectomy. A totally minimally invasive approach (minimally invasive/robot-assisted abdomen and thorax) was performed in 11 centers, while a hybrid approach (robot-assisted abdomen and open thorax) was used as standard access in one. In one center, a total open approach was preferred. Only two of the centers performed pyloric drainage routinely, while eight (62%) placed a feeding jejunostomy. Regarding postoperative strategies, all centers routinely placed an NG-tube, the detailed use of which (suction on NG, routine X-ray/CT evaluation), however, varied between the centers (Table 1). Timing of enteral feeding depended on the routine use of a feeding jejunostomy or not as centers using jejunostomy started feeding earlier (POD 0–1) compared with the latter (POD 3–8).

**Table 1:** Participating Nordic gastrointestinal cancer centers

Hospital	Reported yearly esophageal resections	Preferred surgical access in the abdomen/ thorax	Usage of NG (Yes/No)	Continuous suction on NG-tube (Yes/No)	Removal of NG-tube (POD)	Pyloric drainage (Yes/No)	Routine use of feeding jejunostomy (Yes/No)	Start of jejunostomy feeding (POD)	Start of peroral feeding (POD)	Postoperative radiologic evaluation (POD)
*Sweden*										
Gothenburg	25	Totally minimal invasive	Y	N	3	N	Y	1	3	Not performed
Linköping	21	Totally minimal invasive	Y	N	5	N	Y	1	5	4–5
Lund	45	Totally minimal invasive	Y	N	3–5	N	Y	0	5	Not performed
Stockholm	54	Laparoscopy/ robot-assisted thorax	Y	Y	3	N	Y	1	6	3
Umeå	10	Open abdomen/open thorax	Y	N	3	N	Y	1	5	Not performed
Uppsala	25	Totally minimal invasive	Y	N	5	N	N		6	5
Örebro	15	Totally minimal invasive	Y	N	3	N	N		4	3
*Norway*										
Oslo	50	Totally minimal invasive	Y	N	3–5	N	Y	1	7	3
Tromsø	17	Totally minimal invasive	Y	N	7	N	Y	1	7	6
Trondheim	20	Totally minimal invasive	Y	Y	4	N	N		5	Not performed
*Denmark*										
Copenhagen	110	Robot-assisted abdomen/open thorax	Y	Y	7	Y	N		8	6
Odense	65	Totally minimal invasive	Y	Y	4–5	N	Y	0	5	Not performed
*Finland*										
Oulu	18	Totally minimal invasive	Y	N	5	Y	N		5	5

POD, Postoperative day; NG, Nasogastric.

Randomization and completion of the eCRF is up and running and the inclusion pace is in line with the projections. We aim to complete inclusion during 2024.

## DISCUSSION

This is the first adequately powered RCT investigating the safety of omitting NG tube in the immediate postoperative period after esophagectomy for cancer. The study protocol and logistics are working well without detrimental adverse effects related to the study design. The pretrial survey across Scandinavian UGC centers demonstrated variations in perioperative patient care strategies.

The use of minimally invasive techniques varied across the Nordic UGCs with 85% performing totally laparoscopic or robot-assisted procedures while the remaining centers performed hybrid or open procedures. Minimally invasive surgery may be associated with less pain, less blood loss, shorter length of stay, and even improved survival compared with open surgery.^21^^,^^22^ Furthermore, fewer major postoperative complications and major pulmonary complications after minimally invasive esophagectomy have been found in a recent 5-year survival analysis of an RCT by Nyutens et al.^23^ We have included stratification for center in the randomization in order to obtain well balanced groups in this regard. According to the pretrial survey, a median of 22.5 esophagectomies were performed annually at the Nordic UGCs ranging from 10 to 110 procedures. Standardized surgical procedures and perioperative management protocols are implemented in Nordic UGCs to reduce the potential consequences of complications. However, the pretrial survey across 13the study centers demonstrated apparent differences in the choice of surgical approach and postoperative care across the institutions. Interestingly, the survey also demonstrated high adherence to use of postoperative NG tube use for decompression, although without adequate evidence to support this practice. We chose not to interfere with current perioperative routines outside of the study interventions and this has facilitated swift inclusion and a high level of acceptance of all collaborators in this trial. We believe that this pragmatic approach to the study design will improve generalizability of the findings and applicability to every day practice outside the typically stringent protocols of RCTs.

## Credit author statement

Jakob Hedberg (Conceptualization, Data curation, Formal analysis, Funding acquisition, Investigation, Methodology, Project administration, Resources, Software, Supervision, Validation, Visualization, Writing—original draft, Writing—review & editing), Magnus Sundbom (Conceptualization, Investigation, Methodology, Writing—review & editing), David Edholm (Conceptualization, Investigation, Methodology, Writing—review & editing), Eirik Kjus Aahlin (Conceptualization, Investigation, Methodology, Writing—review & editing), Eva Szabo (Conceptualization, Investigation, Methodology, Writing—review & editing), Fredrik Lindberg (Conceptualization, Investigation, Methodology, Writing—review & editing), Dag Tidemann Førland (Conceptualization, Investigation, Methodology, Writing—review & editing), Jan Johansson (Conceptualization, Investigation, Methodology, Writing—review & editing), Joonas H. Kauppila (Conceptualization, Investigation, Methodology, Writing—review & editing), Michael Hareskov Larsen (Conceptualization, Investigation, Methodology, Writing—review & editing), Per Löfdahl (Conceptualization, Investigation, Methodology, Writing—review & editing), Tom Mala (Conceptualization, Investigation, Methodology, Writing—review & editing), Gjermund Johnsen (Conceptualization, Investigation, Methodology, Writing—review & editing), Lars-Bo Svendsen (Conceptualization, Investigation, Methodology, Writing—review & editing), Magnus Nilsson (Conceptualization, Investigation, Methodology, Project administration, Resources, Supervision, Writing—original draft, Writing—review & editing), Mats Lindblad (Conceptualization, Investigation, Methodology, Writing—review & editing), Pernilla Lagergren (Conceptualization, Investigation, Methodology, Project administration, Writing—review & editing), Oscar Åkesson (Conceptualization, Investigation, Methodology, Writing—review & editing) and Michael Achiam (Conceptualization, Data curation, Formal analysis, Investigation, Methodology, Supervision, Writing—original draft, Writing—review & editing)

## Ethics and consent to participate

This paper has been performed in accordance with the declaration of Helsinki.

## Author contributions

All co-authors who have all contributed significantly, read and approved the final manuscript.

## Financial support

This study was supported by the Swedish Cancer Foundation (CAN 2021/1086) to J.H.

## Conflicts of interest

The authors declare that they have no conflict of interest.
